# Aflatoxin exposure in a population of HIV patients at risk of hepatocellular carcinoma North-Central, Nigeria

**DOI:** 10.4314/ahs.v23i2.9

**Published:** 2023-06

**Authors:** Pantong Davwar, Paul David, Lucius Imoh, Mary Duguru, Kefas Zawaya, Yop Tsok, Atiene Sagay, Edith Okeke

**Affiliations:** 1 Jos University Teaching Hospital; 2 Federal Teaching Hospital Gombe

**Keywords:** Liver Cell Carcinoma, Aflatoxin B1, HIV

## Abstract

**Background:**

Aflatoxin B1causes damage to the DNA by the alkylation of bases and P53 mutation. Exposure to this mycotoxin is associated with the development of liver cancer. Measures to reduce grain and cereal contamination have been a focus however, the effects of these measures are still lagging behind and exposure continues to occur even in populations at risk of developing liver cancer.

**Objective:**

To quantify aflatoxin B1 exposure in a population of HIV infected patients with and without HCC.

**Method:**

This was a cross-sectional study among 196 patients with HIV and or HCC. We evaluated the exposure to aflatoxin B1 using the Aflatoxin M1 metabolite by ELISA on urine samples.

**Results:**

A total of 196 participants consisting of 163 (83.2%) HIV positive and 28 (14.3%) HCC. Mean age is 46.64±10.8 years. The median aflatoxin (IQR) aflatoxin M1level is 177.3(112.5-272) pg/ml. Only 8(4.1%) of the participant had no exposure to aflatoxin B1. The median (IQR) aflatoxin for fibrosis score ≥ 13kpa (178.7(112.9-286.8) pg/ml) VS < 13kpa (173.5(107.9-250.4)), p = 0.046.

**Conclusion:**

There is high prevalence of aflatoxin B1 exposure in this population. Concerted efforts must be put in place to mitigate exposure because of the potential effects of short- and long-term exposure to aflatoxin.

## Introduction

The fungi *A. flavus* and *A. parasiticus* are the predominant aflatoxins producing fungi in West Africa. Aflatoxins contaminate grains and cereals, which are staple food in this region.

This happens mainly due to the tropics' warm, humid climate, which supports the fungus growth and proliferation. Contamination of the staple foods can occur before or after harvest. Out of the over twenty known aflatoxins, four types are particularly harmful to humans. These are aflatoxin B1, B2, G1, and G2.^1^,^2^ It is known that aflatoxin B1 (AFB1) is the most toxic, causing damage to the DNA by the alkylation of bases and P53 mutation.^3^ Thus, AFB1 has been implicated in the pathogenesis of HCC, accounting for 4.6-28.2% of the global hepatocellular carcinoma (HCC) burden.^4^,^5^

Following ingestion, aflatoxins are rapidly absorbed, metabolized by the liver, and the metabolites such as aflatoxin M1(AFM1) which is a 4 hydroxy derivative of AFB1 is excreted in milk and urine. Measurement of the metabolite AFM1 in urine, is an index of dietary AFB1 exposure.^6^–^8^ It has been demonstrated that HCC patients with exposure to this potent carcinogen tend to have 4.5 times more aflatoxins than controls.^3^ The interplay of AFB1 with other risk factors for HCC in the setting of HIV in Nigerians is yet to be determined. Aflatoxins have been classified as class 1 carcinogens by the International Agency for Research on Cancer (IARC). This is the same class as the hepatitis B virus. Both aflatoxin and hepatitis B are present in epidemic proportions in Sub-Saharan countries and are independent risk factors for HCC.

Synergistically, they lead to a 30-fold increased risk of HCC than when they occur alone.^5^

In Nigeria, the high burden of Hepatitis B Virus infection with a national prevalence of 12%.^9^ It is expected that with high consumption of staple foodstuff contaminated by the aflatoxin B1-producing fungus, the synergistic interaction of HIV and AFB1 will present a cauldron for HCC.

There is evidence to show that HCC is promoted by HIV infection due to acceleration of the disease process and promotion of fibrosis of the liver in people living with HIV (PLHIV)^10^–^12^. HIV is also associated with poor survival in HCC. The role of HIV in HCC is related to the degree of immunosuppression that occurs in HIV patients. Recent studies have shown a role for Aflatoxin in promoting HIV infection to AIDS and its role in increased levels of HIV viral load. 13 Patients with higher viral loads tend to have higher levels of aflatoxin biomarker in their blood than those with lower viral loads. Higher viral load is associated with decreased immunity. 14 Reduced free radical scavengers such as vitamins A and E associated with HIV may predispose to more severe pathologic effects of aflatoxin in these individuals. 15

There is a high burden of HIV, HBV infection, and high levels of aflatoxin in Nigeria, it is plausible that there may be synergistic effects of these three agents in predisposition to HCC. However, the role of aflatoxin in HCC among HIV-infected patients has not been clearly defined. Therefore, this study seeks to investigate this relationship by assessing aflatoxin exposure in a population of HIV-infected patients with and without HCC.

## Methods

This study was carried out at the Jos University Teaching Hospital (JUTH), a tertiary institution located in Jos East Local Government Area of Plateau State, North Central Nigeria. The study population included adults aged 18 years or more, diagnosed with HCC, HIV, or both. HCC was diagnosed based on radiologic criteria using a triple-phase CT scan recommended by the AASLD 2018 guidelines. When patients have the characteristic arterial enhancement in the arterial phase and washout in the portal and venous phases at CT scan, they are confirmed to have HCC. Pregnant women and patients with liver cirrhosis or decompensated chronic liver disease due to other causes were excluded from the study. All confirmed HIV patients already on HAART were eligible to be recruited into the study.

A total of 196 participants were seen at the medical out-patient department, accident and emergency unit, in-patient wards, and the AIDS Prevention Initiative in Nigeria supported HIV clinics of the Jos University Teaching Hospital (JUTH), who met the inclusion criteria were recruited into the study. Relevant clinical data and history were obtained for all participants using a prepared case report form and from patients' and medical records. Laboratory results such as hepatitis B, hepatitis C, full blood count, clotting profile, AFP, ESR were obtained from patients record. All participants had an Abdominal Ultrasounds. Those patients with HCC were diagnosed based on radiologic criteria using a triple-phase CT scan recommended by the AASLD 2018 guidelines.

Urine samples were collected from all participants and stored at -80oC for seven months until analysis. According to manufacturer specifications, laboratory analysis of the urine Aflatoxin M1 level was analysed using the Helica biosystems ELISA kit (Cat. No. 991AFLM01U-96). The frozen samples were allowed to thaw to room temperature and debris or precipitate in the urine sample were removed by decantation. The urine specimens were diluted 1:20 with distilled water and analysed in single measurement. All participants had a fibroscan done to determine liver fibrosis status using a medium probe fibroscan Echosens France.

### Statistical analysis

Data was stored using Excel and analysed with SPSS version 20. Quantitative continuous variables were summarized using median (IQR) and non-uniformly distributed quantitative variables as median with range, while categorical variables were expressed as proportions using percentages. The Kruskal Wallis test was used to compare aflatoxin concentration between the three groups. The chi-square test was used to test the significance of association between categorical variables. A p-value of < 0.05 was considered statistically significant in all cases, and the confidence interval was set at 95%.

## Results

There was a total of 196 study participants in this study, out of which 28(14.3%) were HCC patients while 163(83.2%) were HIV positive without HCC and 5(2.6%) had both HIV and HCC. Thirty-eight of the studied participants were positive for the hepatitis B surface antigen. Table 1 below shows the demographic distribution of the study population. There was a total of 126 females accounting for 64.3 % of the studied group. The mean age of the participants is 46.6±10.79; table one below shows the age distribution and demographic characteristics.

**Table 1:** Showing demographic characteristics of the studied population

Variable	Frequency n=196	Percent%
**Diagnosis**		
HIV	163	83.2
HCC	28	14.3
HIV and HCC	5	2.6
**Age group(years)**		
<30	8	4.1
30-49	110	56.1
≥50	78	39.8
Mean±SD	46.64±10.79	
**Sex**		
Male	70	35.7
Female	126	64.3
**Marital Status**		
Married	132	63.3
Widowed	38	19.4
Single	18	9.2
Separated	5	2.6
Divorced	3	1.5
**Education**		
None formal	26	13.3
Primary	55	28.1
Secondary	57	29.1
Tertiary	58	29.6
**Occupation**		
Student	6	3.1
Trading	8	4.1
Artisan	9	4.6
Farming	22	11.2
Unemployed	28	14.3
Civil/ public servant	35	17.8
Business	51	26.0
Others	37	18.9

The median (IQR) AFM1 level in the studied population was 177.3(112.5-272) pg/ml. Among those with HCC, the median (IQR)concentration of aflatoxin was 171.8(91.3-314.7) pg/ml. All HCC patients had aflatoxin biomarker detected in their urine samples. In contrast, those who were only HIV infected had a median (IQR) AFM1 concentration of 178.7(120.2-257.9) pg/ml. The group with HIV and HCC had a median (IQR) concentration of 153.40(101.9-239.3). There was no difference between the AFM1 concentration of the three groups K=0.235 p=0.889.

Only 8 (4.1%) participants did not have any detectable AFM1 in their urine; none had HCC. There was no relationship between gender, alcohol consumption, cigarette smoking, and aflatoxin levels. There was no correlation between aflatoxin concentration and fibrosis score r=-0.035 p=0.625. The median CD4 count for all HIV participants is 165.0(91.00-336.0), with no correlation between CD4 count and AFM1 concentration.

## Discussion

In this study, we demonstrated high ongoing aflatoxin exposure in a population of HIV-infected patients and HCC patients. Aflatoxin M1 is a marker of acute and ongoing exposure to aflatoxins in the past 72 hours.^16^ There was a uniform exposure to aflatoxins among the HCC patients in their urine samples among the sampled population. This ongoing exposure to AFB1 which is classified by IARC as a class 1a carcinogen among patients who already have the feared outcome of aflatoxin exposure calls for the attention of regulatory bodies. Some studies have demonstrated a relationship between urinary aflatoxin levels and dietary exposure. 6,17 Although we only quantified acute exposure from urine samples in this study and not diet, it is plausible that the source of exposure may be dietary. Compared to other studies in other regions of Africa with a similar aflatoxin burden, our study showed a similar high burden of exposure. For instance, a study from Egypt among infants with Kwashiorkor had reported 80% 18 of positive detects, while in Kenya, AFM1 was detected in 78% of samples 19, 71.75% in Uganda 20, and 99% in Tanzania 21 . Another author in western Nigeria also documented a prevalence of 99% of AFM1 in urine samples.^22^

Our study focused on a population of HIV and HCC patients. Exposure to aflatoxin among them should be considered a piece of essential clinical information. This is because aflatoxin has been implicated in the pathogenesis of HCC via its genotoxic effect in causing a mutation in the TP53 gene encoding the P53 tumor suppressor gene.^23^ Studies have demonstrated that chronic exposure to aflatoxin increases the risk by many folds for developing HCC.^24^

It is interesting to note that this critical risk factor for HCC was present in all HCC patients studied. Although our study did not demonstrate a causal relationship between aflatoxin presence and HCC, the mere fact that an implicated carcinogen is present in the body fluids of this patient is an important finding that should alert public health institutions. Secondly, HIV is an immunosuppressive disease condition, and aflatoxin has been demonstrated and implicated as an immunosuppressant.^25^–^28^ Hence the presence of both HIV and Aflatoxin may worsen the immunosuppressed state of the individual. Our study did not demonstrate this finding as there was no correlation between either CD4 count and viral load with Aflatoxin exposure. These may however be a reflection of the effect of antiretroviral therapy. Our HIV cohort of patients have been on antiretroviral therapy for a variable length of time and may have had both virologic and immune reconstitution, which might have accounted for the lack of correlation between the aflatoxin and CD4 count.

Although, in our study we did not find a significant correlation between the degree of hepatic fibrosis and urinary concentrations of AFM1 and the mechanism of aflatoxin-induced liver damage is not thought to include cirrhosis formation, it is important to observe that having both cirrhosis and aflatoxin exposure will be a double hit in the HCC pathway. These further buttresses the point that measures to reduce aflatoxin exposure are required to be put in place to reduce exposure and the risk of HCC progression. The current evidence suggests that aflatoxin may be a risk factor for liver cirrhosis. A study carried out in the Gambia suggested that aflatoxin may be implicated in cirrhosis development. 29 This finding has been corroborated by another study from Guatemala.^30^ Also, a recent meta-analysis by Mekuria et al. showed that there is a significant increase in the risk of liver cirrhosis in individuals with significant aflatoxin exposure.^31^

Several other studies have demonstrated a synergistic role between aflatoxin exposure and chronic hepatitis B virus (CHBV)infection in the pathogenesis of HCC, and furthermore, evidence is beginning to be available to demonstrate that this synergy may be via a cirrhotic pathway.^23^,^24^,^32^ In this hyperendemic environment for CHBV infection, this synergistic activity is an important factor in HCC manifestation. In our studied population of predominantly HIV patients, the prevalence of hepatitis B virus infection based on the surface antigen-positive status in the population was 18%. This implies that this group of patients are at an increased risk of HCC, as suggested by previous data to be about sixfold higher than those with either risk factor in isolation.^33^ This synergy should bolster the argument that in a setting where HCC is endemic, measures to reduce either risk factor or a combination of the two acting in concert should be intensified. In their study in 1999, Sun et al came to the conclusion that exposure to aflatoxin in the setting of CHBV infection increases the risk of developing HCC 3.3 fold 95%CI 1.2-2.8, especially in those with detectable AFM1 in their urine samples.^34^ This was further corroborated by researchers from Taiwan who also reported the synergy between CHBV and aflatoxin in HCC occurrence.^35^ If measures to control viral hepatitis are handled in concert with measures to reduce aflatoxins, this will aid in the fight against hepatic carcinogenesis.

In the future, researching the relationship between dietary exposure, acute exposure, and chronic exposure to aflatoxin and hepatocarcinogenesis will be a worthwhile topic to pursue. Also, further studies to demonstrate the percentage of HCC that occur as a result of aflatoxin exposure will also be an important tool in hand for the purpose of advocacy to stem the immediate and long-term effect of aflatoxin exposure on the liver and other systems of the body. Our study was limited by our inability to quantify the dietary exposure among these patients to be able to demonstrate a relationship between types of diet on the concentration of aflatoxin. Other studies that corroborate our findings that were done in Nigeria and elsewhere have demonstrated a relationship between dietary aflatoxin consumption and levels of AFM1 in urine and breast milk in varying concentrations.^7^,^36^

The very high prevalence of AFB1 metabolites in this studied population is an important pointer to the public health risk this fungus poses. Concerted efforts must be put in place to mitigate exposure to aflatoxin because of the potential effects of both short and long-term exposure to aflatoxin. Enhanced public health interventions are required to reduce the burden of exposure.

## Figures and Tables

**Table 2:** Comparing the median AFM1ng/ml in study participants (Sex, Alcohol use, Tobacco use, and Fibrosis score)

Variable	Median (IQR)	U-test	p-value
**Sex**		3978.000	0.294
Male	194.80 (121.30-254.95)		
Female	140.60(104.62-282.10)		
**Alcohol**		1886.00	0.812
Yes	165.05(110.55-326.48)		
No	4.65(121.68-262.50)		
**Tobacco use**		695.000	0.117
Yes	126.00(106.10-184.80)		
No	194.80(121.92-284.95)		
**Fibroscan**		3495.500	0.758
≤13 Kpa	181.40(121.30-250.90)		
>13 Kpa	147.40(94.10-315.35)		

***IQR=interquartile Range     U-test= Mann Whitney U***

**Figure 1:**
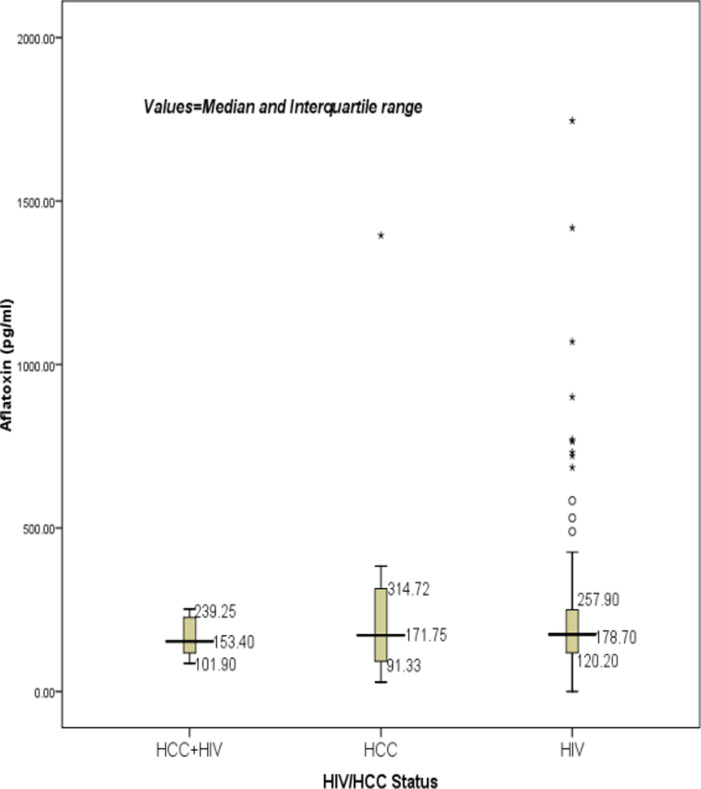
Showing a box plot comparing the mean AFM1 and also shows the mean and IQR
